# Modeling Alzheimer's Disease with Induced Pluripotent Stem Cells: Current Challenges and Future Concerns

**DOI:** 10.1155/2016/7828049

**Published:** 2016-05-24

**Authors:** Weiwei Zhang, Bin Jiao, Miaojin Zhou, Tao Zhou, Lu Shen

**Affiliations:** ^1^Department of Neurology, Xiangya Hospital, Central South University, Changsha, Hunan 410008, China; ^2^Department of Human Genetics, Emory University School of Medicine, Atlanta, GA, USA; ^3^State Key Laboratory of Medical Genetics, Central South University, Changsha, Hunan 410008, China

## Abstract

Alzheimer's disease (AD) is the most prevalent type of dementia and its pathology is characterized by deposition of extracellular *β*-amyloid plaques, intracellular neurofibrillary tangles, and extensive neuron loss. While only a few familial AD cases are due to mutations in three causative genes (APP, PSEN1, and PSEN2), the ultimate cause behind the rest of the cases, called sporadic AD, remains unknown. Current animal and cellular models of human AD, which are based on the A*β* and tau hypotheses only, partially resemble the familial AD. As a result, there is a pressing need for the development of new models providing insights into the pathological mechanisms of AD and for the discovery of ways to treat or delay the onset of the disease. Recent preclinical research suggests that stem cells can be used to model AD. Indeed, human induced pluripotent stem cells can be differentiated into disease-relevant cell types that recapitulate the unique genome of a sporadic AD patient or family member. In this review, we will first summarize the current research findings on the genetic and pathological mechanisms of AD. We will then highlight the existing induced pluripotent stem cell models of AD and, lastly, discuss the potential clinical applications in this field.

## 1. Introduction

Since Alzheimer's disease (AD) was first diagnosed by Dr. Alois Alzheimer in 1906 [1], it has become the most prevalent neurodegenerative disease overall. Over 30 million people had suffered from AD worldwide before 2010, and the count number is estimated to double every 20 years to reach 66 million in 2030 and 115 million in 2050 [2] (http://www.alz.co.uk/research/statistics; accessed October 9, 2012).

Clinically, AD is characterized by gradual memory loss and a progressive learning disability and inability to carry out daily tasks. The main pathological hallmarks of AD are thought to be the deposition of extracellular senile plaques composed of insoluble *β*-amyloid (A*β*) peptide, the formation of intracellular neurofibrillary tangles, and the loss of cholinergic neurons in the basal forebrain, amygdala, hippocampus, and cortical area. However, only the A*β* and the abnormal truncated and hyperphosphorylated tau hypotheses cannot fully explain all of the symptoms of AD. Indeed, various antiamyloid drugs succeeded in lowering the A*β* levels in the brain but failed to slow down the cognitive decline in the treated patients [3, 4]. Additionally, antitangle drugs, which target the kinases and activators involved in the hyperphosphorylation of tau (including the GSK-3 inhibitors, Tideglusib and the methylthioninium chloride tau aggregation inhibitor, Rember) were successful in phase II clinical trials [5] but showed imprecise efficacy in larger phase II trials.

Due to AD's multifactorial and heterogeneous features, its ultimate etiology of AD is not thoroughly understood. While mutations of Presenilin 1 (*PSEN1*), Presenilin 2 (*PSEN2*), and amyloid precursor protein (*APP*) account for most of the early-onset familial AD cases [6, 7], the etiology of the remaining 95% sporadic AD patients is complicated, which is due to various factors including aging, gender, education, and genotype of apolipoprotein E (*ApoE*) [8]. Therefore, there is a pressing need for the emergence of new technologies and models reflecting the progression of AD in patients, confirming the disease pathology, and predicting novel or optimal therapeutic strategies.

Since its creation in 2006 by Yamanaka groups, induced pluripotent stem cell (iPSC) is considered as a potential tool for modeling neurodegenerative diseases [9]. By forced expression of certain genes, including Oct 3/4, Sox2, Klf4, and c-Myc, patients' specific somatic cells are reprogrammed towards their pluripotent state. In this manner, iPSCs are generated artificially and regain the ability to convert into any cell type of the three germ layers: mesoderm, ectoderm, and endoderm. Several preclinical studies, by modeling both familial and sporadic AD, have established promising methods to gather insights into the exact cellular mechanisms, potential therapeutic strategies, and personalized treatments for AD. Here we summarize the current research on the pathogenesis and iPSC-based models of AD and highlight the potential future application of these cells.

## 2. Genetics and Pathology of AD

Given the fact that most AD cases are sporadic and that the disease occurs at an old age, an increasing evidence indicates that the underlying cellular or molecular pathological process may start early and progress throughout one's life. The early-onset, familial AD (FAD) accounts for less than 5% of all AD sufferers, and the late-onset, sporadic AD (SAD) affects the remaining 95% [10] (http://www.molgen.vib-ua.be/ADMutations/). FAD and SAD appear to share the same clinical and pathological process in a way that both types of AD patients exhibit progressive dementia clinically, extracellular A*β* plaques, and intracellular accumulation of phosphorylated tau protein. In general, major achievements of understanding to AD came from the study of the familial AD and mostly from FAD patients with disease-causing mutations.

Genetic factor is considered to be among main contributors to the risk of AD. Mutations in disease-causing genes and disease-risk genes have been identified and linked with either early-onset AD (EOAD) or late-onset AD (LOAD) (Table 1). Usually, EOAD is inherited in an autosomal dominant manner, and by linkage analyses three rare forms of EOAD have been identified to be linked to their causative genes which include one that encodes for the amyloid precursor protein (APP) and two coding for presenilin, PSEN1 and PSEN2. Approximately 50% FAD patients carry mutations in the three causative genes. Among them, mutations in PSEN1 that represent the majority comprise the majority (approximately 70–80%) of the mutations in EOFAD, followed by APP mutations (15–20%) and mutations of PSEN2 accounting for less than 5% [10]. The amyloid cascade hypothesis demonstrates the underlying targets of the three causative genes. In central nervous system (CNS), the APP protein functions as a neuron surface receptor and participant in neurite growth, neuronal adhesion, and axonogenesis. Physically, the APP protein is cleaved by *α*-, *β*-, and *γ*-secretase at three major sites, respectively. The *α*-secretase (mainly ADAM10, a disintegrin and metalloproteinase 10) mediated cleavage reduces the production of A*β*, while *β*-secretase (mainly BACE I, *β* site APP-cleaving enzyme I) and *γ*-secretase lead to A*β* production [11, 12]. PSEN1 and 2 are transmembrane protein components of the *γ*-secretase complex involved in A*β* production during APP processing. The A*β* clearance pathway includes Neprilysin, IDE (insulin-degrading enzyme), ECE (endothelin-converting enzyme), and ACE (angiotensin-converting enzyme) [13, 14]. Imbalance between production and degradation of A*β* (e.g., the mutations of APP, PSEN1, and PSEN2) results in its accumulation and aggregation in the brain. The consequences of A*β* accumulation include a series of abnormal cellular responses such as the formation of intracellular neurofibrillary tangles (NFTs) made of abnormal truncated and hyperphosphorylated tau [15, 16], microglial and astrocytic activation, inflammatory response, oxidative stress, mitochondrial dysfunction, and at last neuron loss. Tau is a microtubule-associated protein with function of promoting microtubule assembly and stability that may also be involved in the establishment and maintenance of neuronal polarity, axonal transport, and neurite outgrowth, although there are no known tau mutation in AD. In the AD brain, the principal hallmark of tau pathology is the formation of paired helical filaments (PHFs) and NFTs. Tau hyperphosphorylation is a potent inducer of tau pathology because hyperphosphorylated tau displays an increased propensity to form PHFs. It is possible that A*β* peptides that have initially accumulated in the AD brain could activate some tau kinases to promote tau phosphorylation through insulin or wnt pathway [17, 18]. Among these, GSK3*β* is identified to be able to phosphorylate tau at several sites to form PHFs in neurofibrillary tangles distributed in AD brain [19]. In some PSEN1 mutation cases, GSK3 also became active with the existence of A*β* peptide [20]. On the other hand, tau is also a substrate for various proteases. Truncations of tau protein at aspartic acid 421 (D421) and glutamic acid 391 (E391) residues by several caspases are associated with NFTs in the brains of AD patients [21, 22]. In vitro A*β* treatment produces a 17 kDa fragment (tau 45–230), and overexpression of it induces neuronal apoptosis [23]. Additionally, Calpains, thrombin, and cathepsins are also involved in tau truncation apart from caspase [24–26]. However, more tau fragments found in AD brain are not well investigated and their production and impact remain to be identified.

Due to the multifactorial and heterogeneous nature of AD, genetic counseling of SAD is empiric and relatively nonspecific. It is often speculated that SAD is the combined action of unknown environmental factors and multiple susceptibility genes. Among them, frequent variations of apolipoprotein E (*APOE*) are the only well-documented association with SAD. APOE is a component of several lipoproteins consisting of 3 isoforms determined by cysteine-to-arginine substitutions at positions 112 and 158 of the amino acid sequence [27]. Individuals with heterozygous APOE *ε*4 are 4 times more likely to develop AD while homozygous for APOE *ε*4 are 8 times relative to individuals without APOE *ε*2 and APOE *ε*3 allele [28]. In CNS, APOE is thought to facilitate clearance of A*β*, and the APOE *ε*4 allele seems to have the lower ability to clear A*β* resulting in a high risk of developing AD. APOE *ε*4 is also identified with smaller gray matter volumes and accelerated brain aging [29, 30].

With the application of Genome-Wide Association Study (GWAS) since 2005, next generation whole exome (WGS), and whole-genome sequencing (WES) to identify common and rare variations, a series of genes or locus are proposed to increase the risk of AD. These include the identified common risk variation of* CR1*,* CLU* [31, 32],* PICALM* [32],* BIN1* [33],* HLA-DRB5/DRB1* [34],* CD2AP*,* MS4A*,* ABCA7*,* CD33* [35, 36],* INPP5D*,* MEF2C*,* SLC24A4-RIN3*,* CASS4*,* NME8*,* ZCWPW1*,* PTK2B*,* CELF1*, and* SORL1* [34]. Their functions vary from brain development, guiding neural plasticity, cytoskeletal organization, cell apoptosis, and lipid metabolism to A*β* uptake and microtubule cytoskeleton interaction (Reviewed in [37]). While these frequent variations are responsible for risk of AD, rare variation detected from WES/WGS might have larger effect sizes than the common variations. A rare variant of Triggering Receptor Expressed on Myeloid Cells 2 (*TREM2*) gene, the rs75932628 (R47H) mutation was confirmed to increase the age of onset in LOAD patients [38]. Further studies on the* TREM2*-associated risk of AD indicated that it is the recessive loss-of-function mutations in* TREM2* that were responsible for early-onset dementia [39]. Generally speaking, TREM2 is expressed by microglial cells in CNS and was found to be presented with amyloid plaques in the brain of AD mice, suggesting that TREM2 may play a role of A*β* clearance. The presence of* TREM2* R47H variant was also confirmed in population from French [40], Spanish [41], Catalan [42], and Belgian [43]; however, in a study involving 1133 patients and 1157 subjects from China, the R47H variant was not detected [44]. In our study with 360 AD cases and 400 controls of Chinese population, the rs201280312-T (A130V) variant was detected in two of the AD cases [45], suggesting the genetically heterogeneous nature of TREM2 mutations.

Other rare variations were identified in genes coding for Netrin receptor,* UNC5C* [46], phospholipase D3 (*PLD3*) [47], ATP-binding cassette transporter (*ABCA1*) [48], and disintegrin and metalloproteinase domain-containing protein 10 (*ADAM10*) [49, 50], although their functional participation in AD occurrence needs further investigation. Elucidating the genetic contribution is a major concern in understanding SAD while there is neither animal models nor proper cell models of SAD to modeling SAD in a dish.

## 3. iPSCs Reprogramming and the Basis of Alzheimer's Disease Modeling

Both embryonic stem (ES) cells and induced pluripotent stem cells (iPSCs) have the ability to self-renew and differentiate into all three germ layers, thereby endowing us the possibility of reconstructing all types of cells, tissues, and even organs. However the applications for human ES (hES) are limited by several challenging problems such as allogeneic immune rejection, potential tumor formation, and ethical issues concerning the utility of human embryo [51, 52]. Derived from human fibroblast, iPSC was first generated by Yamanaka groups in 2007 [53]. With the similar ability of differentiation with ES cells, but without the concerns of immune rejection problems or ethical issues, iPSC soon gained worldwide attention.

### 3.1. Introduction of iPSCs Technology

The iPSCs were first generated from mouse fibroblasts through the retroviral-mediated introduction of four transcription factors (OCT4, SOX2, KLF4, and c-MYC) by Takahashi and Yamanaka in 2006 [9]. They found that forced expression of the four extrinsic factors was sufficient to return somatic fibroblasts into a pluripotent state within a few weeks. As soon as a year after this breakthrough, the technique was applied to human fibroblasts [53, 54]. The induced pluripotent stem cells showed similar colony morphology, gene expression, cell surface marker expression, and the ability to self-renew and differentiation as embryonic stem cells (ESC). Since then, more combinations of transcription factors emerged, such as forced expression of OCT4, SOX2, NANOG, and LIN28 mediated via lentiviral vector to reprogram human fibroblasts into an undifferentiated state [55]. One of the concerns for iPSCs methodology is that the insertion of retrovirus vectors into human genome might become a potential threat to troublesome changes such as tumor-genesis. To avoid shortcomings brought by viral vector interaction, nonintegrating viruses have been applied for generation of the iPSCs, including Adenovirus [56] and Sendai Virus [57–60]. However, these methods are either of low efficiency or technological immaturity. Thus, more alternatives for nonintegrating methods were invented such as transfection miRNA for transcription factors [61], episomal plasmids, three oriP/EBNA plasmids (a kind of plasmid vector that may express for a long period of time) harboring either an Oct4, Sox2, Nanog, and Klf4, an Oct4, Sox2, and SV40 large Tantigen, or a c-myc and Lin28 combination. This way, human foreskin fibroblasts were reprogrammed into iPSCs as soon as 20 days after transfection [62].

Efforts were also directed at improving the reprogramming efficiency as the original method of reprogramming by Yamanaka achieved an efficiency of only ~0.02% at ~30 days after retroviral transduction [53] and the mRNA expression method mentioned above was able to raise the efficiency to 1.4% within 20 days [61]. It has been found that by shifting the culture condition to 5% O_2_ and adding valproic acid into the cell culture medium, the efficiency could be increased to 4.4% [61]. miRNA has been believed to be another promising factor that could increase the reprogramming efficiency with or without Yamanaka factors since some miRNAs are upregulated in both iPSCs and hESCs. For instance, with the presence of four Yamanaka factors, miR-302b and/or miR-372 could increase the efficiency of reprogramming in MRC5 and BJ-1 fibroblasts from 10- to 15-fold compared with the four factors alone [63], while expression of miR302/367 only could transform ~10% of the BJ-1 fibroblasts into iPSCs after 12–14 days after infection [64].

In addition to seeking safer reprogramming factors and improving the reprogramming efficiency, searching for the proper cell sources for reprogramming represents another important strategy. Although skin fibroblasts are considered as the traditional, classical cell source for iPSCs generation, one must undergo a rather invasive procedure for donating samples. The collection of cells from other lower yield sources can be far less invasive, including the collection of mononuclear cells from peripheral blood [65], or hair follicles [66], or even the exfoliated renal epithelial cells from urinary sediments [67]. However, due to the inherent inefficiency of iPSC generation, a large amount of somatic cells is required. Furthermore, the culture potential of the primary cells often varies according to the donors' ages, physical conditions, and long-term drug use. Consequently, it is urgent to decipher the primary underlying cause of the differences between various cell sources and to find an easier method for isolating enough somatic cells in the least invasive manner.

### 3.2. Application of iPSC in Alzheimer's Disease

In light of the important benefits conferred by their self-renewal and multidirectional differentiation capacity, iPSCs are valued in the context of regenerative medicine and disease modeling, especially for neurodegenerative disorders. Indeed, despite the limitations imposed by the low-efficiency and time-consuming nature of the reprogramming process, iPSCs remain a relevant tool to study the fundamental etiology of neurological diseases and perform high-throughput drug screening for CNS disorders. In fact, several neurological diseases have been modeled using iPSCs, such as monogenic disorders and versions of complex diseases caused by known mutations have been modeled by iPSCs. These disorders include, among others, Parkinson's disease (PD) with SNCA, PINK1, PARK2, GBA1, and LRRK2 mutations [68–70], amyotrophic lateral sclerosis (ALS) with TDP43 mutation [71], Huntington's disease (HD) with HTT mutation [72], and Spinal muscular atrophy (SMA) with SMN1 mutation [73]. Similarly, AD is another slow progressing disease with a poorly understood etiology and a lack of efficient therapeutic strategies. Therefore, it is of the utmost importance for the AD patients' unmet clinical needs that we identify suitable, disease-relevant cell models to solve these problems.

iPSCs can be directionally differentiated into neurons using a specific array of protocols. First, neural stem/progenitor cells are generated from iPSCs with the presence of the neuroectoderm inducer, retinoic acid [74–76]. A similar outcome can also be achieved by inhibiting the bone morphogenetic protein (BMP) and the transforming growth factor-*β* (TGF*β*) superfamily signal transduction pathways [77, 78], both of which are capable of directing epidermal or mesodermal differentiation. Then, these neural stem cells could be further exposed to certain growth factors to direct differentiation into specific neuronal subtypes. To model AD, induced cholinergic neurons can be generated using a combination of Compound E, 2S-2-N-propanamide (Calbiochem), and Compound W, 3,5-bis(4-nitrophenoxy)benzoic acid, to activate specific intracellular signaling pathways targeting the repressor element 1-silencing transcription factor (REST) and its corepressor (CoREST) [79, 80]. These induced neurons are further selected by specific markers expressed on the endogenous neurons. Upon transplantation into animal models of neurodegenerative diseases, these neurons function well and contribute to the recovery of several neurological deficits.

**Figure 1:**
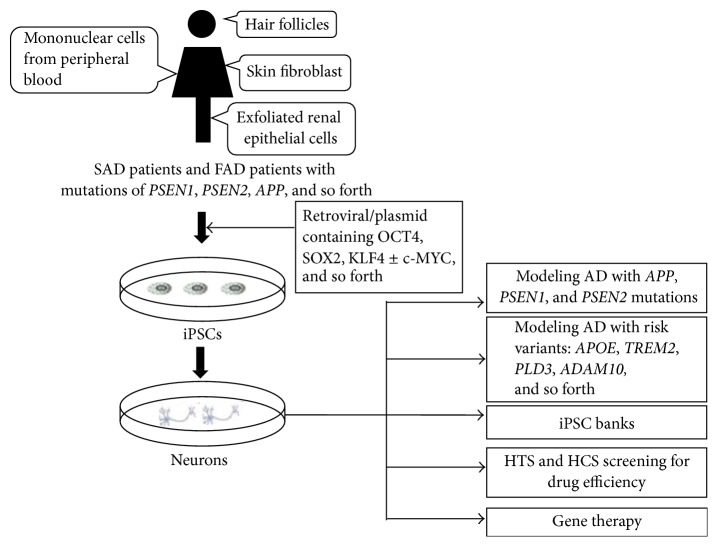
The generation of iPSCs and iPSC-derived neurons from various sources of somatic cells and application of iPSC in Alzheimer's disease.

In general, isolated somatic cells undergo a series of reprogramming and neural differentiation procedures to generate a large number of induced AD patient-specific neurons for both research and transplantation purposes (Figure 1). However, there are some hurdles to be overcome before attaining the stage of clinical application. First, even though iPSCs and induced neurons retain the original patients' specific genome, some random DNA alterations and epigenetic changes cannot be avoided during either the reprogramming or the differentiation processes [81]. The potential alterations in DNA splicing or gene expression may induce clonal heterogeneity within the iPSCs and result in cellular functional changes. Second, neuroglia cells participate in the induction of immune responses and A*β* peptide clearance in AD pathogenesis, and the participation of astrocytes and microglia cells may have an important influence on our understanding and interpretation of the figuring out of the AD-specific cellular phenotypes and drug efficiency. Actually, GWAS analyses have yielded a pattern of common cellular pathways involved in AD patients carrying certain risk variants. In the future, it will not be enough to induce the formation of cholinergic neurons only. Instead, three-dimensional human neural cell culture models will be essential for accurate AD modeling [82]. Third, before reaching the stage of clinical application, it will be essential to determine the optimal and safest iPSC generation and neuron differentiation protocols to use. Indeed, some protocols using integrative viruses and the culture media or feeder cell layers containing animal components constitute potential health threats due to the potential for unwanted immune responses and tumor-genesis.

## 4. Specific Cellular Phenotypes and Processes in the iPSC-Based Models of AD

Modeling AD using iPSCs was initiated from the modeling of familial cases with mutations in disease-causing genes including* APP*,* PSEN1*, and* PSEN2*. Until now, five out of eight publications reported reprogramming of iPSCs-derived cholinergic neurons from patients with FAD (summarized in Table 2), indicating that modeling AD using iPSCs is still in its infancy. However, these studies, which are seeking to find AD-specific unique cell phenotype and AD-related cellular processes, appear as the first step in gaining insights into the genetic contributions to AD.

### 4.1. FAD Disease Modeling with iPSC

iPSCs have been generated from patients with several mutations of* PSEN1*,* PSEN2,* and* APP* by five groups. Two groups analyzed the production of A*β* peptides and the accumulation of phosphorylated tau protein. Both* PSEN1* A246E- and* PSEN2* N141I-expressing mutant neurons showed an increased ratio of A*β*42 to A*β*40 compared with control neurons, but the ratio in iPSCs lines was very low, indicating that the secretion of A*β* peptides varies during differentiation. In addition, no accumulation of tau protein was observed in this type of FAD-derived neurons [80]. However, the* PSEN1* A246E mutant was further analyzed during the differentiation and observed an increase in the ratio of A*β*42 to A*β*40 in both fibroblasts, neural progenitor cells (NPCs) and early neurons [83], which is somewhat conflicting with the result of Takuya Yagi groups [80].

Induced neurons that carry a duplication of APP exhibited a higher level of A*β*40 but not A*β*42. In fact, the A*β*42 and A*β*38 levels were completely lower than the detection range of the assay [84]. Through fluorescence-activated cell sorting, researchers were able to isolate a more than 95% pure culture of induced neurons. Purified neurons also exhibited higher levels of phosphorylated tau (Thr 231) and active GSK-3*β*. Compared with control neurons, RAB5-positive early endosomes were enlarged in the neurons from patients with duplication of* APP*, suggesting that early endosomes may regulate APP processing to result in the increased level of phospho-tau, neurofibrillary tangles, synaptic loss, and apoptosis. Muratore et al. found that iPSCs and neurons harboring the* APP* (V717I) mutation showed a twofold increase in the production of A*β*42 and a slight increase in A*β*40 [85]. The A*β*38 level and the calculated A*β*38/40 ratio were also significantly increased compared with control neurons [85]. Furthermore, FAD neurons secreted a lower ratio of APPs*α*/APPs*β*. The APPs*β* production showed a 1.4-fold increase compared with controls [85], suggesting that the V717I mutation may primarily alter the initial epsilon site of cleavage within APP. In another study in patients with APP E693Δ mutation [86], the A*β* oligomers accumulated in the iPSCs-derived neurons and astrocytes with APP E693Δ. The hall markers of ER stress and oxidative stress, including BiP, cleaved caspase-4, PRDX4-coding antioxidant protein peroxiredoxin-4, and ROS levels, were also increased in the FAD neurons. Therefore, there was a possibility that the intracellular A*β* oligomers may provoke an antioxidant stress response resulting in increased ROS levels. These results supported the hypothesis that oxidative stress participates in the pathogenesis of AD.

### 4.2. SAD Disease Modeling with iPSCs

Primary cells from SAD patients have also been used for reprogramming studies and were mostly compared with iPSC originating from cells donated by FAD patients. By researching into the SAD case, Hossini et al. were able to draw an AD-related protein interaction network composed of* APP* and GSK3*β* among others [87]. In Israel et al.'s study, relative to nondemented controls, both iPSCs and neurons generated from mutation of the* APP* gene and SAD patients showed elevated levels of A*β* peptides, hyperphosphorylation of tau, and GSK3*β*. While neurons from only one of two SAD patients exhibited increased levels of intracellular A*β* aggregates, similar to the cells derived from the* APP*-E693Δ FAD patients. It is possible that some underlying de novo acquired genes may also participate in the pathogenesis of SAD cases, reflecting the inherent variability of iPSCs. A recent gene expression study in neurons derived from an 82-year-old SAD patient revealed significant gene expression changes between primary cells and induced neurons [87]. The iPSCs technique offers an opportunity to study the underlying molecular events leading to SAD without interference of environmental contributions, allowing the identification of novel AD-associated networks of regulated genes. However, one concern related to the heterogeneous nature of SAD is that the iPSCs-derived neurons from AD patients without inheritance need to expand until cells are produced to enable statistically meaningful analyses.

### 4.3. Using iPSC-Derived Models to Screen Novel Drugs for AD

Novel treatments targeting the amyloid cascade, APP processing, and ER stress have been tested on the iPSC-derived models of both FAD and SAD. Some of them exhibited significant efficiency on the cell-based models and may become candidate drugs to cure AD patients in the future. *γ*-secretase inhibitors were first screened in iPSCs and neurons carrying* PSEN1* A246E and* PSEN2* N141I mutations [80]. In the presence of compound E, a potent *γ*-secretase inhibitor, both A*β*42 and A*β*40 decreased sharply in a dose-dependent manner in FAD neurons. Another *γ*-secretase substrate, the Notch intracellular domain, was also inhibited in a dose-dependent manner, suggesting that both* PSEN1* and* PSEN2* iPSCs-derived neurons respond to the *γ*-secretase inhibitor treatment. In Muratore et al.'s study, both FAD neurons with* APP* V717I mutation and SAD neurons responded to DAPT, another *γ*-secretase inhibitor, and the induced neurons exhibited an inhibited production of A*β*38, 40, and 42 when treated with 5 *μ*m DAPT for 48 hours [85]. A*β* antibody that binds and sequesters the A*β* peptides was able to prevent the increase in the total tau levels in* APP* V717I neurons, suggesting a crosstalk between the amyloid cascade and tau hyperphosphorylation in the AD brains [85]. However, in the clinical trials, although the antibody succeeded at lowering the levels of A*β* peptides, it failed at slowing down the progression of the cognitive impairment. Docosahexaenoic acid (DHA) has been reported to improve ER stress or to inhibit ROS generation [88]. In* APP*-E693Δ neurons, DHA could significantly decrease the BiP protein, cleaved caspase-4, and peroxiredoxin-4 levels, as well as reduce the ROS production, ER stress, and oxidative stress markers. As a consequence, the neurons with an* APP*-E693Δ mutation survived longer after DHA treatment for 16 days compared with the SAD and control neurons [86]. Considering that the levels of A*β* oligomers, which trigger the ER and oxidative stress, remained unchanged, this indicates that DHA treatment may be considered as a symptom relief invention but not as a preventive/curative therapy. Taken together, these results show that iPSCs-derived models allow screening for proper treatment strategies under specific individual genomes.

## 5. Challenges and Concerns

The potential applications of the reprogramming technology provide a promising approach to generate accurate human cell models of neurodegenerative disorders. To a certain extent, it now becomes possible to aim at recapitulating AD “in a dish.” While iPSCs-derived cell models allowed the identification of factors associated with the disease phenotypes and the screening of various novel potential therapies, for example, the *β*- and *γ*-secretase inhibitors on the basis of the amyloid cascade hypothesis, there are still more discoveries to be made by using iPSC-derived models of AD (Figure 1).

### 5.1. Investigation of Novel Mutations in* APP*,* PSEN1*, and* PSEN2*

So far the iPSCs generated from fibroblasts with several FAD mutations, including* PSEN1* A264E, L166P and M146L,* PSEN2* N141I,* APP* V717I, E693Δ, and duplication of* APP* are summarized in Table 2. Compared with neurons induced from control fibroblasts, FAD presenilin and* APP* mutant neurons exhibited not only an increased A*β*42/40 ratios, an elevated level of phosphorylated tau protein, and an increased activated GSK3*β*. Furthermore, they also displayed abnormal endosomes, indicating a novel dysregulated pathway alongside of APP processing and tau phosphorylation. Therefore, more iPSCs with additional FAD mutations should be investigated to confirm the existing conclusions and help identify other underlying mechanisms behind the disparate phenotypes of the patients.

### 5.2. Investigation of AD Risk Variants Identified by GWAS, WES, and WGS

Whole-genome sequencing and large scale genome-wide association studies aimed at elucidating the factors that result in SAD has brought to light a variety of rare and frequent variants that may predispose or to protect from AD. However, the processing of these data represents a major challenge. For example, screening for these variants in diverse disease populations is already planned, but the related underlying molecular and biochemical mechanisms as well as the impact of the different genetic variants on the neuronal phenotype and AD risk still remain an unsolved puzzle. Actually, several common cellular pathways are associated with variations identified as GWAS, such as inflammation and immune response, endocytosis, and lipid metabolism. The application of the iPSCs technology to study those potential AD risk variants maybe an efficient way to identify the ultimate impact of these genes on the pathology of SAD, to distinguish between the real and the false positive variations and to find out novel pathways associated with the pathogenesis of AD.

### 5.3. Construction of the iPSCs Bank for AD

Although cord blood bank has been initiated in many countries, given the expensive procedure and the limited availability at the moment of birth only, iPSCs bank would be a promising way alternative to the cord blood bank to restore and recycle cells from patients with different phenotype and mutations because the iPSCs technology could generate a self-renew, stable progenitor population. Public banks of diseased fibroblasts from patients with genetic mutations responsible for certain neurodegenerative disorders already exist. The existence of these banks allows for categorizing sporadic cases and familial cases with different mutations for personalized medicine purposes, investigating the typical phenotype of each individual's unique genetic background, and may ultimately provide a potential treatment means for regenerative medicine.

### 5.4. Novel Drug Testing: High-Throughput Screening (HTS) and High Content Screening (HCT)

Since iPSCs could retain the patients' genotype and enable us to recapitulate AD in a dish, neurons derived from the disease-specific iPSCs have been used to test several candidate drugs, such as the *γ*- and *β*-secretase inhibitors and A*β* antibody. The five studies based on iPSCs-derived models of AD patients harboring the mutations of PSEN1, PSEN2, and APP point mutation as well as APP duplication, respectively, all report decreasing A*β* peptide levels in iPSCs-derived neurons treated with a *γ*-secretase inhibitor, an A*β* antibody, and DHA. The reduction in phosphorylated tau and GSK-3*β* levels was observed in the neurons treated with a *β*-secretase inhibitor. Likewise, the nonsteroidal anti-inflammatory drug (NSAID) sulindac sulfide has been proposed to become one of the novel strategies for AD therapy by inhibition of the A*β* production [89]. The stem cell-derived neurons expressing wild-type* PSEN1* treated with NSAID exhibited a decrease in A*β*42 level. However the therapeutic effect was absent in cells harboring the* PSEN1* L166P mutation [90]. These studies constitute the primary attempts at screening potential treatment strategies in human genotype- or disease-specific cells. Using iPSCs, HTS, and HCT could allow for rapid analysis for thousands of compounds and disease hallmarks, as well as various cellular contexts affecting drug efficiency, or chemical toxicity, for example. Indeed, by accessing the induced neurons from a cohort of patients and controls in a 96-well format, researchers could rapidly analyze a substantial number of drugs and chemicals for endpoints such as A*β* peptides levels or phosphorylated tau levels. Using the HTS technique as a prescreening method would streamline the time-consuming preclinical animal studies and potentially reduce the number of failing clinical trials. After target drugs or compounds identification using HTS, HCS would enable the subsequent analysis of relevant cellular signals and pathways. Highly efficient screening based on iPSC-derived neurons could become a routine drug discovery pathway.

### 5.5. Gene Therapy and iPSCs Transplantation

The potential iPSCs-based regeneration medicine and gene therapy on AD include gene correction and iPSCs induced neurons transplantation. Gene correction has already been conducted in HD by using homologous recombination in the iPSCs stage, leading to normalized pathogenic HD signaling pathways, including cadherin, TGF-*β*, BDNF, and caspase activation in neural stem cells [72]. On the basis of the cellular endogenous recombination mechanism, zinc-finger nucleases (ZFNs) and tal-effector nucleases (TALENs) are emerging as engineered nucleases usable to modify individual genomes [91, 92]. First, a wild-type nucleotide sequence binds into the FOK1 nuclease fused to arrayed domains triggering a DNA double-strand break. Then, the endogenous recombination machinery emerged induces DNA homologous recombination and nonhomologous end-joining. Finally, the disease-causing mutations are corrected into the wild-type genomic sequence. Genetically corrected iPSCs or induced neurons transplantation in CNS is still at the stage of animal testing. In 1985, researchers successfully transplanted embryonic cholinergic neurons into an AD rat model. The procedure resulted in memory improvement, suggesting that cholinergic cells transplantation may induce functional recovery in the rodent brains [93]. With the generation of the first iPSCs in 2006, the idea of autologous transplantation emerged with the advantage of reducing immunoreactions usually associated with heterologous transplantation. A recent study on the autologous transplantation of iPSC-derived dopamine neurons in a cynomolgus monkey (CM) PD model demonstrated that after iPSC-derived dopamine neurons injection on one side of midbrain, the CM exhibited motor improvement on the transplanted side without a need for immunosuppression. This study indicated a progression that is a step closer to human clinical applications [94]. However, safety is an essential problem concerning insertional mutagenesis and tumorigenesis prior to clinical use. The amount of induced neurons required for functional improvement is another hurdle, an improvement in the iPSCs/neurons generation protocols is indispensable to get equivalent or greater neuron survival.

After all, the iPSCs technology provides a potential therapy for monogenic disorders, as to AD patients with mutations in* APP*,* PSEN1*, and* PSEN2*, shedding light on ultimate therapy of FAD by correcting these mutation. This strategy also holds great promise for complex diseases like SAD. For example, correcting the mutations of risk genes selectively enables a direct comparison between the iPSC lines derived from WT and mutant cells under the same genome circumstance, significantly reducing the intrinsic genome variability existing in different patients.

## Figures and Tables

**Table 1:** Causative and risk variants of AD.

Gene	Variant	Effect allele frequency	Odds ratio	Function	AD-related pathways
APP	—	—	—	A*β* peptide precursor, neurite outgrowth, adhesion, and axonogenesis	APP processing, produce A*β*
PSEN1	—	—	—	Component of *γ*-secretase complex that cleaves APP into A*β* fragments	APP amyloidogenic pathway, cleaves APP
PSEN2	—	—	—	Component of *γ*-secretase complex that cleaves APP into A*β* fragments	APP amyloidogenic pathway, cleaves APP

Frequent variants					
APOE	—	—	—	Component of lipoproteins, transports lipids and cholesterols, mediates synaptogenesis, synaptic plasticity, and neuroinflammation	A*β* clearance
CR1	rs6656401	0.197	1.18	Bind C3b and C4b, and moderate the activity of the complement system	A*β* clearance
BIN1	rs6733839	0.409	1.22	Participant in Clathrin-mediated endocytosis, intracellular trafficking, apoptosis, and interacting with the microtubule cytoskeleton	Mediate tau toxicity
CD2AP	rs10948363	0.266	1.10	Cytoskeletal organization, endocytosis	Mediate tau toxicity
EPHA1	rs11771145	0.338	0.9	Mediate brain development, particularly axonal guidance	Immune response, neural development
CLU	rs9331896	0.375	0.86	extracellular chaperone, inhibits formation of amyloid fibrils by APP	A*β* clearance
MS4A6A	rs983392	0.403	0.9	Signal transduction	unknown
PLCALM	rs10792832	0.358	0.87	Clathrin-mediated endocytosis	A*β* clearance
CD33	rs3865444	0.307	0.94	Inhibition of cell activity	A*β*42 uptake
HLA-DRB5– HLA-DRB1	rs9271192	0.276	1.11	Histocompatibility antigen, peptide antigen binding	Immune response
PTK2B	rs28834970	0.366	1.10	Induce long term potentiation in hippocampus	Synapse function and neural development
SLC24A4 and RIN3	rs10498633	0.217	0.91	Calcium transport, brain and neural development	Neural development
DSG2	rs8093731	0.017	0.73	Component of intercellular desmosome junctions	unknown
INPP5D	rs35349669	0.488	1.08	Regulate cell proliferation and survival	Immune response
MEF2C	rs190982	0.408	0.93	Participant in hippocampal-dependent learning and memory by suppressing the number of excitatory synapses,and neuronal development and distribution	Neural development, synapse function
NME8	rs2718058	0.373	0.93	Spermatogenesis, ciliary functions	unknown
ZCWPW1 and NYAP1	rs1476679	0.278	0.91	Epigenetic regulation (ZCWPW1); brain and neural development (NYAP1)	Neural development
CELF1	rs10838725	0.316	1.08	mRNA splicing	unknown
FERMT2	rs17125944	0.092	1.14	Cell adhesion, cell shape and Wnt signaling pathway	Mediates tau toxicity
CASS4	rs7274581	0.083	0.88	cell adhesion and cell spreading	Cytoskeleton and axonal transport
SORL1	rs11218343	0.039	0.77	Lipoprotein uptake, APP trafficking to and from Golgi apparatus	APP trafficking
ABCA7	rs115550680	0.09	1.79	lipid metabolism, phagocytosis of apoptotic cells	A*β* clearance

Rare variants					
ADAM10	Q170H and R181G	—	—	Constitute and regulate *α*-secretase activities	APP nonamyloidogenic pathway
APP	rs63750847	0.0045	0.24	Inhibit *β*-secretase activities	APP processing
TREM2	rs75932628	0.0063	2.26	A*β* clearance	Immune response
UNC5C	rs137875858	0.0003298	2.15	Increase susceptibility to neuronal cell death	Inflammatory response
PLD3	rs145999145	0.003077	2.1	APP trafficking and cleavage	APP processing
AKAP9	rs144662445	0.0006298	2.75	unknown	unknown
	rs149979685	0.000432	3.61		

**Table 2:** Human somatic cell reprogramming-based neuronal models of Alzheimer's disease.

Disease	Genetic defect	Outcome	Drug test	Reference
Azheimer's disease	PSEN1 A264E; PSEN2 N141I	Increase secretion of A*β*1–42 in neurons with mutations	*γ*-secretase inhibitors	Yagi et al., 2011 [80]
	Duplication of *APP*; Sporadic	Increase secretion of A*β*1–42 and phosphorylated tau (Thr231) in neurons with mutations	*β*-secretase inhibitors	Israel et al., 2012 [84]
	APP E693Δ; APPV717L; Sporadic	Increase of intracellular A*β* olimgo in neurons with APPE693Δ;	DHA	Kondo et al., 2013 [86]
	Asymptomatic and symptomatic APP V717I	Increase secretion of A*β*1–42 and A*β*1–38 in neurons with mutations	A*β* antibody	Muratore et al., 2014 [85]
	PSEN1 A246E; PSEN1 M146L	Gene expression differences between neurons with mutations of *PSEN1* and controls	no	Sproul et al., 2014 [83]
	Sporadic	Changes in gene expression as well as the inducible subunits of the proteasome complex associated with AD in AD-iPS derived neuronal cells	*γ*-secretase inhibitors	Hossini et al., 2015 [87]
